# Body Image Perception, Weight Status and Health Behaviours Among African Immigrant Women Living in Italy: A Cross-Sectional Comparative Study

**DOI:** 10.3390/nu18152456

**Published:** 2026-07-27

**Authors:** Stefania Toselli, Emanuela Gualdi-Russo, Luciana Zaccagni

**Affiliations:** 1 Department for Life Quality Studies, University of Bologna, 47921 Rimini, Italy; stefania.toselli@unibo.it; 2 Department of Neuroscience and Rehabilitation, Faculty of Medicine, Pharmacy and Prevention, University of Ferrara, 44121 Ferrara, Italy; luciana.zaccagni@unife.it; 3 Center for Exercise Science and Sports, University of Ferrara, 44123 Ferrara, Italy

**Keywords:** immigrants, obesity, Mediterranean diet, physical activity, body image dissatisfaction, women, acculturation

## Abstract

**Background/Objectives**: Body image is influenced by body weight, sociocultural norms, and weight-related behaviours. Migration has been associated with changes in body ideals, potentially through acculturation processes. This study investigated body image perception, weight status, and related behavioural and psychological factors among a group of African immigrant women living in Italy, comparing them with an Italian comparison group. **Methods**: This cross-sectional study included 131 women (60 African immigrants; 71 Italians). Body image perception was assessed using the Thompson and Gray silhouette scale. BMI-based weight status was derived from self-reported weight and height and compared with perceived weight status. Body dissatisfaction was calculated as the discrepancy between perceived and ideal body image. Additional measures included appearance-related anxiety (PASTAS), self-esteem, life satisfaction, physical activity, and adherence to the Mediterranean diet. **Results**: Immigrant women had significantly higher body weight and BMI (*p* < 0.001) and differed in distribution across weight status categories (*p* = 0.001). Although body dissatisfaction did not differ significantly between groups, immigrant women selected larger ideal body figures (*p* < 0.001). They also showed lower agreement between BMI-based and perceived weight-status, and higher appearance-related anxiety on the PASTAS Non-Weight subscale (*p* < 0.05). No significant differences were observed in self-esteem, life satisfaction, or adherence to the Mediterranean diet. Immigrant women were more likely to be sedentary (38.3% vs. 4.2%) and reported lower levels of physical activity (active: 25.0% vs. 54.9%). **Conclusions**: Compared with the Italian comparison group, African immigrant women in this study showed a greater prevalence of overweight and obesity, preference for larger body ideals, lower agreement between BMI-based and perceived weight status, and lower physical activity levels. Given the cross-sectional design and the characteristics of the recruited samples, these findings should be interpreted as associations rather than evidence of causal relationships. They support the need for culturally sensitive strategies to improve weight awareness and promote healthy lifestyles among African immigrant women.

## 1. Introduction

Obesity is a major public health concern and a well-established risk factor for cardiovascular, renal, hepatic, respiratory, musculoskeletal, and neuropsychiatric diseases, as well as type 2 diabetes and several forms of cancer. Although the global prevalence of obesity has increased substantially since the end of the twentieth century, recent evidence suggests that this trend has stabilized or slightly declined in some European countries, including Italy [1]. Therefore, understanding the factors that influence body weight and weight-related behaviors remains a public health priority.

Body image represents one of the key psychological determinants of weight-related behaviors. The way individuals perceive and evaluate their body size and shape may influence dietary habits, physical activity, and weight-control practices. Misperception of body weight or limited awareness of obesity-related health risks may foster a false sense of security regarding health status, thereby encouraging the continuation of unhealthy lifestyles [2,3]. Conversely, body dissatisfaction may motivate individuals to adopt healthier behaviors and engage in weight-management efforts, although excessive dissatisfaction may also be associated with psychological distress and maladaptive weight-control behaviors [2,4].

Body image ideals are significantly influenced by sociocultural factors, resulting in substantial variations across populations. Traditionally, many African societies have associated larger female body sizes with prosperity, health, fertility, social status, and beauty [5,6,7]. However, growing evidence suggests that these preferences are changing. Recent studies conducted in several African countries have reported an increasing preference for slimmer body types, reflecting the diffusion of globalized Western beauty ideals and broader sociocultural changes linked to globalization and urbanization [6,8,9]. This transition appears particularly relevant among women, who generally experience greater body image concerns and higher levels of body dissatisfaction than men [10,11].

Migration may further accelerate changes in body ideals through the combined effects of acculturation and globalization following intercultural contact [7,8,12]. These processes involve ongoing interaction between migrants and the host culture, potentially shaping beliefs, values, health behaviors, and aesthetic preferences. Consequently, African migrants living in Western countries may gradually adopt thinner body ideals that align more closely with those prevalent in the host country. Previous studies in the United States, Europe, and Australia have documented shifts in body-size preferences among African-origin populations after migration [7,13,14,15,16]. Nevertheless, the extent of these changes may be influenced by several factors, including ethnicity, gender, age at migration, length of residence in the host country, socioeconomic status, and degree of cultural integration [7,17].

Italy represents a particularly interesting context in which to investigate these issues. Immigration to Italy has increased substantially over the last two decades [18], with foreign-born residents accounting for approximately 9% of the total population in 2024 [19]. Among African communities, migrants originating from North African countries such as Morocco, Egypt, Tunisia, and from sub-Saharan countries such as Senegal and Nigeria constitute some of the largest groups [20]. The growing demographic relevance of these populations highlights the need to better understand cultural factors that may affect health behaviors, body weight perception, and health outcomes.

Existing evidence suggests that overweight and obesity are highly prevalent among immigrant populations in Italy. Previous national data indicated that women originating from North Africa and sub-Saharan Africa exhibit some of the highest rates of overweight and obesity among immigrant groups [21,22,23,24]. In particular, studies conducted in Italy have shown that North African immigrant women present high levels of adiposity and relevant differences in body image perception compared with both Italian women and women living in North Africa [15]. Moreover, the risk of overweight and obesity appears to increase with age and duration of residence in Italy, suggesting a possible influence of post-migration environmental and behavioral factors [17,24]. This pattern is consistent with evidence suggesting that the initial health advantage often observed among migrants may decline over time, possibly reflecting assimilation processes and changes in lifestyle after migration [25]. At the same time, recent global evidence points to heterogeneous obesity trends, including a stabilization or decline in obesity prevalence in several European countries and a continuing increase among women in many low- and middle-income countries, particularly in sub-Saharan Africa [1]. These evolving epidemiological patterns further emphasize the importance of re-examining body image ideals and weight perceptions among African immigrant women.

Despite the growing literature on obesity and migration, there remains limited contemporary evidence regarding body image dissatisfaction among African immigrant women living in Italy. More broadly, recent evidence suggests that migration-related experiences may contribute to body image dissatisfaction and eating-related concerns among migrant populations [26]. In particular, little is known about how body image perceptions are shaped by the interaction between migration experiences and current obesity trends in the host population. This gap is particularly relevant because body image perception may influence both the recognition of overweight/obesity and the adoption of weight-related health behaviors.

Migration may influence not only body image ideals but also health-related behaviours and psychological well-being. Changes in dietary habits and physical activity patterns may occur following migration [27,28], while body image has been associated with indicators of well-being such as self-esteem and life satisfaction [29]. However, little is known about these factors among African immigrant women living in Italy and their potential relationship with body image perception.

Therefore, the primary aim of the present study was to investigate body image perceptions and weight status among African-born immigrant women aged 18 years and older living in Italy. Specifically, we aimed to: (1) compare body image ideals and body image dissatisfaction between a group of African immigrant women and a group of Italian-born women; (2) examine the relationship between body mass index (BMI) and body image perception in the two groups; and (3) explore anxiety related to the appearance of specific body regions. In addition, we assessed selected behavioral, psychological, and health-related indicators, including self-esteem, life satisfaction, physical activity level, and adherence to a Mediterranean dietary pattern. These measures were included to provide a broader understanding of the factors associated with body image perceptions while maintaining a brief and feasible survey protocol. The findings may contribute to the development of culturally sensitive health-promotion and obesity-prevention interventions targeting African immigrant women.

## 2. Materials and Methods

### 2.1. Study Design and Participants

This cross-sectional study was conducted between January 2025 and January 2026 in accordance with the Strengthening the Reporting of Observational Studies in Epidemiology (STROBE) [30] guidelines (Table S1) through an online survey. The study protocol was approved by the Bioethics Committee of the University of Bologna (approval code 0157694; 6 June 2024).

African-born immigrant women were recruited through presentations at intercultural and integration centers, and social cooperatives, as well as through flyers distributed at community centers and Italian-language schools. Italian-born women were primarily recruited among employees and students of the universities of Bologna and Ferrara, via institutional notices linking to the online survey. Recruitment was also carried out through referrals from previous participants. All participants were given detailed information about the study’s objectives and procedures via the participation form, and could complete the questionnaire only after providing written informed consent electronically. Participation was anonymous and voluntary.

At the beginning of the survey, participants were asked to choose whether they wished to complete the questionnaire in Italian or English. All subsequent questionnaires and study questions were presented in the selected language. Standardized instruments were administered using the Italian or the original English versions.

The inclusion criteria were: (i) female sex; (ii) age between 18 and 70 years; and (iii) African-born immigrant or Italian-born, according to the study group. Participants who did not complete the body image assessment, pregnant women, and immigrants who had been in Italy for less than a year were excluded from the study.

A total of 140 women participated in the study. After applying the eligibility criteria, 131 participants were included in the final analyses, comprising 60 African-born immigrant women and 71 Italian-born women.

An a priori power analysis was performed using the G*Power statistical program (version 3.1.9.6; Universität Kiel, Kiel, Germany). Assuming a medium effect size, a significance level of 0.05, and 80% power, the minimum required sample size was estimated to be 128 participants for independent-group comparisons using *t*-tests or analysis of covariance (ANCOVA), and 122 participants for chi-square analyses. The final sample size exceeded these requirements.

### 2.2. Data Collection and Measures

The survey collected socio-demographic information, including place and date of birth, educational level, employment status, marital status, number of children, and years of residence in Italy for immigrant participants.

Participants also self-reported body weight and height. Body mass index (BMI) was calculated as weight (kg) divided by height squared (m^2^), and weight status was classified as underweight, normal weight, overweight, or obese according to World Health Organization criteria [31]. Furthermore, the perceived weight status was assessed through the question: “Please select the weight status that you believe best represents you”. BMI-based weight status derived from self-reported height and weight was distinguished from participants’ self-reported weight status throughout the manuscript.

Body image perception was assessed using the Thompson and Gray Body Image Assessment Scale [32]. Participants selected the silhouette that best represented their current and ideal body sizes. Body dissatisfaction was quantified using the Feel-Ideal Discrepancy (FID) index, calculated as the difference between perceived and ideal body image [33]. Higher absolute discrepancy values indicate greater dissatisfaction. Weight status perception accuracy was assessed using the Feel-weight status minus Actual-weight status Inconsistency (FAI) index [34]. This score was obtained by subtracting the participant’s objectively classified BMI category from the weight status category corresponding to the selected silhouette according to the scale of Thompson and Gray [32]. Unlike the perceived weight-status question (self-reported), which was based on participants’ direct classification of their own weight status, the FAI was derived from the silhouette selected to represent their current body size. Thus, the perceived weight-status question and the FAI represent two complementary but distinct measures of weight-status perception. Positive values of FAI indicate overestimation of one’s own weight status, whereas negative values indicate underestimation. Participants also indicated any previous attempts to lose weight and the methods used.

Anxiety related to physical appearance was evaluated using the Physical Appearance State and Trait Anxiety Scale (PASTAS) [35], consisting of 16 items scored on a five-point scale ranging from 0 (“never”) to 4 (“almost always”). Following the original PASTAS validation, anxiety scores were also analysed separately for the Weight and Non-Weight subscales, each obtained by summing the corresponding eight items. General health perception was assessed through a single-item self-rated health question with five response categories ranging from excellent to poor. Self-esteem was measured using the 10-item Rosenberg Self-Esteem Scale (RSES) [36] according to a four-point Likert scale ranging from ‘strongly agree’ to ‘strongly disagree’, and life satisfaction was assessed using the 5-item Satisfaction With Life Scale (SWLS) [37], that was administered using a 7-point Likert scale ranging from 1 (“strongly disagree”) to 7 (“strongly agree”).

Physical activity was evaluated using the Rapid Assessment of Physical Activity (RAPA), which is a nine-item questionnaire that assesses aerobic activity (RAPA 1), strength training, and flexibility (RAPA 2). It requires a ‘yes’ or ‘no’ response [38,39].

To assess eating habits, participants were asked whether they considered the traditional African or Mediterranean diet to be healthy, and were also asked to complete a questionnaire: the Mediterranean Diet Adherence Screener. This questionnaire, as described by Sofi et al. [40], yields a total score ranging from 0 to 18. Higher scores indicate greater adherence to the Mediterranean dietary pattern.

No imputation of missing data was performed. Analyses were conducted using available-case analysis, with each statistical procedure based on all available observations for the variables included. Generally, missing data were minimal (<5%), except for some socio-demographic information in the African-born group (educational level: 25%) and dietary and physical activity habits (~10–20%).

### 2.3. Statistical Analysis

Categorical variables are presented as frequencies and percentages, whereas continuous variables are reported as means and standard deviations (SDs). The normality of continuous variables was evaluated using the Kolmogorov–Smirnov test. The internal consistency of the PASTAS Weight and Non-Weight subscales was assessed using Cronbach’s alpha coefficient.

Student’s *t*-tests were used to compare age and stature between immigrant and non-immigrant women. ANCOVA, adjusting for age, was performed only for the number of children, body weight, and BMI because of the significant difference in age between the two groups. For variables violating the assumptions of normality, non-parametric tests were used: the Mann–Whitney U test for independent samples, and the Wilcoxon signed-rank test for paired samples. Associations between categorical variables were assessed using chi-square tests.

Years of residence were analysed both as a continuous variable and after stratification using a 10-year cut-off [17]. RAPA-1 and RAPA-2 responses were analysed both as categorical variables to examine differences in the distribution of participants across response categories, and as ordinal variables to assess their associations with FID.

Agreement between objectively classified weight status (BMI-based weight status) and self-reported weight status was evaluated using Cohen’s kappa coefficient. Spearman’s rank correlation coefficients were calculated to assess the associations between FID scores and physical and mental health variables and behaviours, and between BMI and body image indicators.

All statistical analyses were performed using Statistica for Windows (version 11.0, StatSoft Inc., Tulsa, OK, USA).

Statistical significance was set at *p* < 0.05.

## 3. Results

### 3.1. Sample Characteristics and Weight Status

A total of 60 immigrant women (mean age: 35.0 ± 11.1 years) and 71 non-immigrant women (mean age: 29.5 ± 14.5 years) completed the online questionnaire.

The African women had immigrated to Italy from 14 different countries. The most frequently reported countries of birth were Morocco (33.3%) and Senegal (15.0%), followed by Nigeria (10.0%), Ivory Coast (8.3%), Tunisia (6.7%), Cameroon (5.0%), Egypt (3.3%), and other less represented countries (18.4%). The mean duration of residence in Italy was 11.4 ± 10.5 years. Regarding marital status, one-third of the immigrant women were single, whereas two-thirds were married. One-quarter of the immigrants had no children, 23.3% had one child, 26.7% had two children, and 25% had three or more children. Among non-immigrant women, approximately half were single, and half were married, while only 12.7% had children. Educational attainment differed markedly between the two groups (*p* < 0.001). Among immigrant women, educational levels were relatively evenly distributed across primary (20.8%), lower secondary (29.2%), upper secondary (25.0%), and university education (25.0%). In contrast, the non-immigrant sample was highly educated, with 93.0% of participants holding a university degree and only 7.0% having completed upper secondary education. Employment status also differed substantially between the two groups. Among immigrant women, 83.3% were unemployed, and only 16.7% were employed. By contrast, among non-immigrant women, 40.8% were unemployed, and 59.2% were employed (*p* < 0.001).

Table 1 shows the quantitative characteristics of immigrant and non-immigrant women, along with the results of statistical comparisons between the two groups.

Immigrant women were significantly heavier and had a higher BMI than non-immigrant women. In particular, the mean BMI of immigrant women fell within the overweight range, whereas the mean BMI of non-immigrant women was within the normal-weight range.

The distribution of weight status (Table 2), as determined by BMI, differed significantly between immigrant and non-immigrant women (*p* = 0.001; Cramer’s V = 0.353). In particular, immigrant women showed a higher prevalence of overweight and obesity compared with non-immigrant women (51.7% vs. 21.1%). Regarding perceived weight status, no statistically significant differences were found between the two groups (*p* = 0.08; Cramer’s V = 0.227), although a greater proportion of immigrant women perceived themselves as obese.

The analysis of agreement between perceived and BMI-based weight status revealed notable differences between the groups. Among non-immigrant women, 77.5% of participants correctly classified their weight status, whereas 14.1% underestimated and 8.5% overestimated it. In contrast, among immigrant women, only 50.0% correctly classified their weight status, while 33.3% underestimated and 16.7% overestimated it. The comparison between the two groups showed a statistically significant difference in the distribution of underestimation, accurate perception, and overestimation (*p* = 0.004; Cramer’s V = 0.288), indicating lower accuracy in weight status perception among immigrant women.

These findings were further supported by the Cohen’s Kappa analysis between actual and perceived weight status, which yielded a value of 0.65 (substantial agreement) among non-immigrant women and 0.34 (fair agreement) among immigrant women, indicating a higher level of agreement between perceived and BMI-based weight status in Italian women than in immigrant women.

### 3.2. Body Image Perception

Among immigrant women, silhouette 6 was the most frequently selected feel body image, whereas silhouette 5 was the most frequently selected among non-immigrant women. Regarding ideal body image, silhouette 5 was the most commonly chosen by immigrant women, while silhouette 3 was the preferred choice among non-immigrant women. In both groups, the ideal silhouette was significantly thinner than the perceived silhouette (*p* < 0.001).

Considering the weight status of the silhouettes selected as ideal, the overweight and obese categories were combined because of the very small number of participants selecting an obese silhouette (3.3% of immigrant women). While the normal-weight silhouette was the most frequently selected category in both groups (53.3% among immigrants and 71.8% among non-immigrants), the distribution of ideal silhouette choices differed significantly between the two groups (*p* = 0.008; Cramer’s V = 0.273). Non-immigrant women were more likely to select an underweight silhouette (18.3%), whereas immigrant women more frequently selected an overweight silhouette (28.3%), suggesting a preference for thinner body ideals among non-immigrant women and for relatively larger body ideals among immigrant women.

Table 3 presents the comparison of body image perception indicators between immigrant and non-immigrant women. Unlike the agreement analysis reported in Table 2, which was based on the direct self-reported perceived weight-status question, the FAI was derived from the silhouette selected to represent the participant’s current body size, assessing a distinct aspect of weight-status perception. Immigrant women reported a significantly higher feel figure and ideal figure scores than non-immigrant women, indicating a preference for a larger ideal body size. In contrast, no significant differences were observed between the two groups for FID or FAI scores.

Mean FID scores were similar between immigrant and non-immigrant women and were close to 1 in both groups, indicating a comparable desire to be thinner (FID > 0 in 48.3% of immigrant women and 57.7% of non-immigrant women). Likewise, mean FAI scores were very similar and close to zero, although slightly negative, suggesting an overall accurate perception of weight status with a slight tendency toward underestimation. Among immigrant women, 56.7% of participants had an FAI of 0, indicating an accurate perception of their weight status, whereas equal proportions (21.7%) underestimated (negative FAI) and overestimated (positive FAI) their weight status. Among non-immigrant women, 74.6% had an FAI of 0, 15.5% underestimated their weight status, and 9.9% overestimated it. A trend towards a more accurate perception of weight status was observed among non-immigrant women than among immigrant women. Indeed, 74.6% of non-immigrant women correctly perceived their weight status compared with 56.7% of immigrant women, while underestimation and overestimation were more frequent among immigrant women (*p* = 0.073; Cramer’s V = 0.200). This finding is consistent with the higher Cohen’s Kappa observed among non-immigrant women (0.65) than among immigrant women (0.34), indicating greater agreement between perceived and BMI-based weight status in the former group. Analyzing the PASTAS according to its original structure, the internal consistency of the two subscales was excellent (Cronbach’s α = 0.907 for the Weight subscale and α = 0.930 for the Non-Weight subscale). No significant differences were observed between immigrant and non-immigrant women for the Weight subscale. In contrast, immigrant women reported significantly higher anxiety related to Non-Weight body areas than non-immigrant women (11.8 vs. 9.0, *p* = 0.011). At the item level, immigrant women reported significantly higher anxiety scores than non-immigrant women for several body parts, including the waist (2.54 vs. 1.86), hands (1.61 vs. 1.14), forehead (1.53 vs. 1.17), ears (1.46 vs. 1.06), neck (1.44 vs. 1.04), and wrists (1.39 vs. 0.96). Because these item-level analyses involved multiple comparisons, they should be considered exploratory.

### 3.3. Relationships Between BMI and Body Image Perception

To further examine the relationship between weight status and body image perception, Spearman’s rank correlations were calculated between BMI and body image indicators in immigrant and non-immigrant women (Table 4).

In both groups, BMI was strongly correlated with feel figure and moderately correlated with ideal figure and FID scores. These findings indicate that women with higher BMI perceived themselves as larger, selected larger ideal silhouettes, and reported greater body dissatisfaction. BMI showed no association with FAI scores.

### 3.4. Exploratory Analyses According to Years of Residence and Region of Origin

Exploratory analyses were performed among immigrant women to investigate whether years of residence in Italy were associated with anthropometric, body image, psychological, dietary, and physical activity variables (Table 5). Spearman’s rank correlation analysis showed that years of residence were positively correlated only with age (ρ = 0.403, *p* = 0.002). No significant correlations were observed between years of residence and BMI, feel figure, ideal figure, FID, FAI, self-esteem score, SWLS score, Mediterranean diet adherence score, RAPA 1, or RAPA 2 score (all *p* > 0.05).

When immigrant women were further stratified according to duration of residence (<10 years vs. ≥10 years using the cut-off adopted in a previous study [17], women residing in Italy for less than 10 years reported a significantly thinner ideal body figure in the unadjusted analysis (Mann–Whitney test; *p* = 0.0445). However, this association was no longer significant after adjustment for age in a general linear model (*p* = 0.209), whereas age showed a borderline association (*p* = 0.056). No significant associations were observed for the other anthropometric, body image, dietary, or physical activity variables.

As an exploratory analysis, immigrant women were further stratified according to their region of origin (North Africa, *n* = 27, and sub-Saharan Africa, *n* = 30). Although the two groups did not differ significantly in age, BMI or years of residence in Italy, women from sub-Saharan Africa reported higher levels of physical activity (RAPA 1 score: 4.0 vs. 2.7), whereas women from North Africa had higher FID scores (1.20 vs. 0.24, *p* = 0.047) and tended to report higher life satisfaction scores (SWLS: 26.1 vs. 21.2), although the latter difference did not reach statistical significance (*p* = 0.052). No significant differences were observed for the remaining variables.

### 3.5. Physical and Mental Health

*Weight-control*. No significant differences were observed between immigrant and non-immigrant women in the frequency of weight-control behaviors (*p* = 0.639; Cramer’s V = 0.137). In both groups, the most common response was “sometimes” (58.5% of immigrant women and 57.7% of non-immigrant women), while only a small proportion of participants reported controlling their weight “always” (7.3% and 7.0%, respectively). Similarly, no significant differences were found between immigrant and non-immigrant women in attempts to lose weight (*p* = 0.086; Cramer’s V = 0.283), although immigrant women more frequently reported never having tried to lose weight (46.3% vs. 19.7%). Conversely, non-immigrant women more often reported attempting to lose weight at least sometimes (80.3% vs. 53.7%). Among women who reported attempting to lose weight, the most commonly adopted strategy was reducing food intake or following a diet. Although the distribution of weight-loss methods did not differ significantly between immigrant and non-immigrant women (*p* = 0.099; Cramer’s V = 0.235), immigrant women more frequently reported reducing food intake or dieting as their primary strategy (68.2% vs. 42.1%). In contrast, non-immigrant women more often reported increasing physical activity (29.8% vs. 18.2%) or following a nutritionist’s advice (28.1% vs. 13.6%).
*General Health Perception*. A significant association was observed between immigrant status and perceived health status (*p* = 0.015; Cramer’s V = 0.275). Immigrant women more frequently reported poor or fair health compared with non-immigrant women (24.4% vs. 7.0%), whereas non-immigrant women more often reported very good or excellent health (50.7% vs. 36.6%). The proportion of women reporting good health was similar in the two groups (39.0% vs. 42.3%).
*Global self-esteem*. Mean global self-esteem scores were comparable between immigrant and non-immigrant women (19.9 ± 4.5 vs. 20.6 ± 5.9, respectively; *p* = 0.657). No significant differences were observed between immigrant and non-immigrant women in global self-esteem levels (*p* = 0.192; Cramer’s V = 0.172). The majority of participants in both groups showed balanced self-esteem (78.0% of immigrant women and 64.8% of non-immigrant women). Very low self-esteem was reported by a similar proportion of women in both groups (14.6% of immigrants vs. 15.5% of non-immigrants), whereas high self-esteem was more frequently observed among non-immigrant women (19.7% vs. 7.3%).
*Life satisfaction***.** The mean quality-of-life scores were similar for immigrant and non-immigrant women (22.4 ± 6.4 vs. 23.7 ± 6.6, respectively; *p* = 0.241). Participants were distributed similarly across the seven response categories. Accordingly, no significant differences in life satisfaction levels were observed between the two groups’ frequencies (*p* = 0.777; Cramer’s V = 0.176). However, a higher proportion of non-immigrant women reported being satisfied or extremely satisfied with their lives compared with immigrant women (52.1% vs. 38.1%).
*Dietary habits*. Among immigrant women, 70.7% perceived the African diet as healthy, whereas 80.5% perceived the Mediterranean diet as healthy. Only 11.7% of immigrant women and 4.2% of the non-immigrant group considered the Mediterranean diet to be unhealthy. Mean adherence scores to the Mediterranean diet were similar in immigrant and non-immigrant women (9.3 ± 3.0 vs. 10.2 ± 2.8, respectively; *p* = 0.106). Consistently, no significant frequency differences were observed between the two groups in terms of adherence to the Mediterranean diet (*p* = 0.369; Cramer’s V = 0.171). The distribution of participants across the four adherence categories was similar in the two groups. Low adherence was the most prevalent category among immigrant women, whereas adequate adherence was the most prevalent category among non-immigrant women.
*Physical activity*. The distribution of RAPA 1 categories differed significantly between immigrant and non-immigrant women (*p* < 0.001; Cramer’s V = 0.453) (Table 6). RAPA 1 results showed that immigrant women were less physically active than non-immigrant women. In particular, 38.3% of immigrant women were classified as sedentary compared with only 4.2% of non-immigrant women, whereas 54.9% of non-immigrant women were classified as active compared with 25.0% of immigrant women. Similarly, the distribution of RAPA 2 categories differed significantly between the two groups (*p* < 0.001; Cramer’s V = 0.610) (Table 6). Among women who completed the RAPA 2 questionnaire, immigrant women reported substantially lower participation in strength and flexibility activities than non-immigrant women. Nearly three-quarters of immigrant women (72.7%) reported no such activities (category 0), compared with 16.9% of non-immigrant women. In contrast, the highest level of participation (category 3) was reported by only 5.5% of immigrant women and 43.7% of non-immigrant women.

### 3.6. Relationships Between FID and Behavioural, Physical, and Mental Variables

The results of Spearman’s rank correlation analysis are shown in Table 7.

In both groups, FID scores were significantly and positively correlated with self-reported weight status and BMI-based weight status. Negative correlations were observed between FID and RAPA 1 among immigrant women and between FID and RAPA 2 among non-immigrant women. Overall, these findings suggest that overweight or obese women tended to report greater body dissatisfaction, while higher levels of physical activity were associated with lower body dissatisfaction.

## 4. Discussion

The present study investigated body image perceptions, body size ideals, weight status, and selected health-related behaviours among African-born immigrant women living in Italy, and compared them with those of Italian-born women. The main findings showed that immigrant women had higher BMI, a higher prevalence of overweight and obesity, larger perceived and ideal body figures, lower agreement between BMI-based and perceived weight status, lower physical activity levels, and poorer perceived health. These findings should be interpreted within the broader context of cultural body-size norms, migration-related experiences, socioeconomic conditions, and health-related behaviours rather than as consequences of migration status alone. This caution is particularly important because the two groups also differed in several socio-demographic characteristics and recruitment settings. The higher BMI observed among immigrant women is consistent with previous studies reporting high levels of overweight and obesity among women originating from North African and sub-Saharan African countries and living in Italy [21,22,23,24]. This finding is particularly relevant in light of recent evidence from NCD-RisC showing heterogeneous global obesity trends. There has been a stabilization or slight decline in obesity prevalence in several European countries, including Italy. However, obesity continues to increase among women in many low- and middle-income countries, particularly in sub-Saharan Africa [1]. It is also in line with evidence suggesting that excess weight among migrants may be influenced by post-migration environmental and behavioural changes, including modifications in diet, occupational activity, social conditions, and opportunities for physical activity [17,25]. However, because of the cross-sectional design of this study, it cannot be determined whether these differences developed before or after migration. An important finding concerns the lower agreement between BMI-based and self-perceived weight status among immigrant women. Although immigrant women showed a higher prevalence of overweight and obesity, their perceived weight status did not differ significantly from that of non-immigrant women. Moreover, agreement between BMI-based and perceived weight status was lower among immigrant women than among non-immigrant women. The Cohen’s kappa values indicate that the two assessments show substantial agreement for non-immigrants and only fair agreement for immigrant women. This suggests that immigrant women were less likely to classify their weight status according to BMI-based categories and more frequently underestimated it. From a public health perspective, this is relevant because underestimation of excess weight may reduce awareness of obesity-related health risks and may limit motivation to adopt preventive behaviours [2,3,34]. This pattern was particularly evident among women classified as obese according to BMI-based weight status from self-reported height and weight. In the immigrant group, a high proportion of women with obesity did not perceive themselves as obese (61.5%), suggesting a possible under-recognition of obesity. This finding may be partly related to cultural perceptions of larger body size, which in some African contexts have historically been associated with health, prosperity, fertility, and social status. However, this interpretation should be considered cautiously, given the heterogeneity of the immigrant sample and the absence of direct measures of cultural body-size norms. In addition, BMI-based weight status was derived from self-reported anthropometric data and should therefore be interpreted cautiously.

Body image perception also differed between the two groups. African immigrant women selected larger perceived and ideal body figures than non-immigrant women. This finding is consistent with evidence showing that, in several African contexts, larger female body size may be associated with health, attractiveness, fertility, prosperity, and social value [5,6,7]. However, the positive FID values observed in both groups indicate that, on average, immigrant and non-immigrant women tended to prefer a smaller body size than the one they perceived. Therefore, the larger ideal body size selected by immigrant women should not be interpreted as the absence of body dissatisfaction, but rather as evidence of a different body-image framework. This interpretation is further supported by the analysis of ideal silhouette categories: although a normal-weight silhouette was the most frequently selected ideal category in both groups, immigrant women more often selected overweight silhouettes, whereas non-immigrant women more frequently selected underweight silhouettes.

Taken together, these findings suggest a transitional body-image pattern, in which larger body ideals may persist among African immigrant women while coexisting with some orientation toward smaller body size, as suggested by previous research [6,8,9,12,13,16]. Because migration and acculturation were central to the rationale of the present study, we performed an exploratory analysis according to duration of residence in Italy. Although women residing in Italy for less than 10 years initially reported a thinner ideal body figure, this association disappeared after adjustment for age, suggesting that the observed difference was largely explained by age rather than by duration of residence itself. Therefore, in our sample, years of residence in Italy were not independently associated with body image outcomes. These findings are consistent with recent evidence suggesting that duration of residence alone is insufficient to capture the multidimensional process of acculturation, as individuals with similar lengths of residence may differ substantially in language proficiency, cultural orientation, social networks, and socioeconomic integration [41].

The absence of significant differences in FID between immigrant and non-immigrant women deserves attention. Body dissatisfaction cannot be inferred from BMI alone. Rather, it must be interpreted in relation to the cultural meanings attributed to body size and body shape [4,7,11]. These findings highlight the importance of considering cultural context when interpreting body image.

Appearance-related anxiety was higher among immigrant women for some specific body areas (e.g., waist), suggesting that body image concerns extend beyond body weight [42,43]. However, the underlying reasons were not directly investigated and should be explored in future studies. Weight-control behaviours did not differ significantly between groups. Although immigrant women tended to report fewer previous weight-loss attempts and relied more frequently on dietary restriction than on physical activity or professional advice, these differences were not statistically significant. Therefore, they should be interpreted cautiously. Nevertheless, the overall pattern may suggest different approaches to weight management and different access to structured health-promotion resources.

Perceived health status differed significantly between groups. Immigrant women more frequently reported poor or fair health, whereas non-immigrant women more often reported very good or excellent health. This finding may reflect the combined influence of higher BMI, lower physical activity, socioeconomic vulnerability, migration-related stressors, and possible barriers to healthcare access. Previous studies have suggested that the health advantage often observed among migrants may decrease over time, partly because of social and environmental conditions in the host country [25]. The present findings are consistent with this broader migrant-health framework. In contrast, global self-esteem and life satisfaction did not significantly differ between groups. This suggests that differences in broader indicators of psychological well-being did not accompany differences in body image and weight perception. Because of the cross-sectional design, the observed associations should not be interpreted as evidence of causal relationships. In particular, it cannot be determined whether migration-related experiences influenced body image and health behaviours, whether these characteristics predated migration, or whether they reflect other social and environmental factors associated with the study populations. Future studies should further examine whether these associations vary according to migration history, social integration, family responsibilities, and socioeconomic conditions.

Dietary findings showed no significant difference in adherence to the Mediterranean diet between immigrant and non-immigrant women. Most immigrant women perceived both the African and Mediterranean diets as healthy. These findings should be interpreted with caution, as the dietary assessment was exploratory and based on self-reported information. Dietary counselling should take into account both traditional dietary habits and Mediterranean dietary recommendations, rather than replacing traditional food practices. Such an approach may be more culturally acceptable and may support sustainable dietary changes. The negative correlation between Mediterranean diet adherence and FID among immigrant women was not statistically significant; therefore, it should be considered only as a possible tendency and confirmed in larger samples.

Physical activity represented one of the clearest behavioural differences between groups, with immigrant women showing lower levels of both aerobic activity and strength/flexibility activities. This finding is particularly relevant in light of their higher BMI based on self-reported height and weight, and poorer perceived health reported among immigrant women. Moreover, the inverse association between physical activity indicators and FID suggests that more active women tended to report lower body dissatisfaction. However, because the study was cross-sectional, this association cannot be interpreted causally. Furthermore, the present study did not assess the reasons underlying the lower physical activity levels observed among immigrant women. Therefore, no conclusions can be drawn regarding the factors responsible for this difference. Previous reviews [44,45] have suggested that physical activity among immigrant women may be influenced by a combination of individual, family, community, and environmental factors, including caregiving responsibilities, time constraints, social support, cultural norms, and access to appropriate facilities. However, these factors were not directly measured in the present study and should be investigated in future research.

Overall, the findings indicate that body image and weight-status perception among African-born immigrant women living in Italy reflect the interaction of anthropometric, behavioural, cultural, and socioeconomic factors rather than migration status alone. Future studies based on larger and more representative samples should further investigate the role of acculturation, migration history, socioeconomic conditions, and barriers to healthy lifestyles in shaping body image and health behaviours. Analyses comparing women from North Africa and sub-Saharan Africa suggested some differences within the immigrant sample. However, given the exploratory nature of these analyses and the limited sample size, these findings should be interpreted cautiously and confirmed in larger studies.

Despite these limitations, the present study provides novel evidence on body image and weight perception among African-born immigrant women living in Italy, a population that remains underrepresented in the literature.

### Study’s Strengths and Limitations

The main strength of this study was the use of a consistent methodology to examine body image perceptions, ideals, and weight status in a sample of African immigrant women compared with women from the host culture. Furthermore, the present study collected a broad range of data that provided new and updated indications on the different behaviors and attitudes regarding the physical and mental health of the women examined, investigating their self-esteem, life satisfaction, physical activity level, and diet.

Limitations of the present study concern, first of all, the cross-sectional design of the study, which does not allow for a direct assessment of the direction of causality between the collected variables. Secondly, anthropometric characteristics and the other selected data were self-reported during the interviews, with the consequent limitations associated with self-reporting. Because height and weight were self-reported in the same survey that included body image questions, responses may have also been influenced by social desirability or body image concerns [46]. Previous studies have shown that adults may underestimate their weight and overestimate their height, which could lead to misclassification of BMI (underestimation) [47]. As no validation measurements were available for this study, it was not possible to assess whether reporting accuracy differed between immigrant and Italian participants. Consequently, any possible differential reporting bias between groups cannot be excluded and may have influenced the observed differences in BMI-based weight status and perceived weight status, as well as the calculation of FAI scores. Nevertheless, self-reported anthropometric data are commonly used in large epidemiological surveys and online studies when direct measurements are not feasible. Although participants were free to complete the survey in either Italian or English, no formal assessment of language proficiency was performed. Furthermore, the standardized instruments have not been specifically validated among African immigrant women living in Italy. Therefore, differences in language proficiency or cultural and linguistic differences in the interpretation of some items cannot be completely excluded. Among the positive aspects of the questionnaires administered, it is worth mentioning that the physical activity questionnaire (RAPA) featured several illustrative images of physical activity, which may have favoured the interviewees’ correct responses. Convenience sampling and the different recruitment strategies of the two subsamples may have introduced selection bias. Specifically, the group of Italian women, recruited largely through university-related settings, may not be fully representative of the Italian female population. Moreover, participants who agreed to take part in the study may represent a selected group with greater interest in health, body image, or weight-related issues, introducing a possible volunteer bias. Similarly, immigrant women recruited through intercultural centers, community centers, cooperatives, and Italian-language schools may not be representative of all African immigrant women living in Italy, particularly concerning socioeconomic background, educational level, integration experiences, and health awareness, As regards the comparison between groups, although age-adjusted analyses were performed when appropriate, African-born and Italian-born differed substantially also in educational attainment, employment status, marital status, and family characteristics. These differences reflect the challenges of recruiting community-based migrant populations and may have acted as confounding factors. For this reason, the observed differences should not be interpreted as reflecting immigrant status per se, but rather as the result of a complex interplay between migration background, socioeconomic conditions, the recruitment setting, and health-related behaviours.

Another limitation is that the African-born immigrant group included women from both North African and sub-Saharan African countries. These populations may be culturally heterogeneous in terms of body size ideals, weight perception, and sociocultural meanings attributed to body shape. Although an exploratory comparison between women from North Africa and sub-Saharan Africa identified some differences within the immigrant sample, these subgroup analyses were exploratory, and the sample size was not sufficient to draw firm conclusions regarding regional differences. Therefore, this heterogeneity should be considered when interpreting body-image-related findings. The present findings should not be interpreted as representative of a single African cultural model. However, the selected approach has allowed us to extend the analysis to African nations that are often overlooked in studies focusing on African immigrant groups from a single country or region because of their accessibility. In particular, this study has expanded knowledge on immigrant women from different areas of Africa, including women from 14 different African countries.

Although the study rationale refers to migration and acculturation processes, acculturation was not directly measured. Years of residence in Italy were collected, but this variable cannot fully capture the complexity of acculturation, cultural orientation, language proficiency, or social integration. Therefore, interpretations related to acculturation should be considered exploratory and hypothesis-generating. Future studies with larger samples and longitudinal designs should examine whether duration of residence in the host country is associated with body image perception, weight-status awareness, dietary habits, and physical activity among African immigrant women.

Therefore, despite the limitations mentioned above, the results of this study should be considered as exploratory.

## 5. Conclusions

In this cross-sectional study, African-born immigrant women showed higher BMI-based weight status, a greater prevalence of overweight and obesity, larger perceived and ideal body figures, lower accuracy in weight-status perception, lower physical activity, and poorer perceived health than the Italian comparison group. However, body dissatisfaction did not differ significantly between the two groups, suggesting that body image cannot be interpreted only based on BMI or weight status.

These findings offer insight into the transitional body image context experienced by African immigrant women, where larger body ideals coexist alongside exposure to host-country weight norms and health-related behaviours. However, these findings should be interpreted with caution because of the cross-sectional design, the use of self-reported anthropometric data, and the different socio-demographic characteristics and recruitment settings of the two groups.

From a public health perspective, interventions should promote accurate awareness of overweight and obesity and support healthy lifestyles through culturally sensitive strategies. Health-promotion messages should focus on health, functionality, well-being, physical activity, and chronic disease prevention, rather than on thinness as an aesthetic ideal.

## Figures and Tables

**Table 1:** Characteristics of the sample by immigrant status (African-born sample *n* = 60; Italian-born sample *n* = 71).

Variables	Immigrants		Non-Immigrants		ANCOVA/*t*-TEST	
	Mean	SD	Mean	SD	*p*	Effect Size (95% CI Where Applicable)
Age (years)	35.0	11.1	29.5	14.5	0.040 ^a^	0.615 (0.97 to 10.03)
Children (number)	1.78	1.77	0.27	0.74	<0.001 ^b^	0.220
Weight (kg)	71.2	15.5	60.8	9.3	<0.001 ^b^	0.118
Stature (cm)	165.3	5.9	164.9	6.6	0.718 ^a^	0.064 (−1.79 to 2.59)
BMI (kg/m^2^)	26.07	5.63	22.36	3.15	<0.001 ^b^	0.118

Note: ^a^ Student’s *t*-test. Effect sizes are expressed as Cohen’s *d*, followed by the 95% confidence interval of the mean difference. ^b^ ANCOVA adjusted for age. Effect size is expressed as partial η^2^.

**Table 2:** Distribution of BMI-based weight status and perceived weight status (direct question) according to immigrant status (African-born sample *n* = 60; Italian-born sample *n* = 71).

Weight Status	Immigrants		Non-Immigrants	
	BMI-Based	Perceived	BMI-Based	Perceived
Underweight	6	3	5	4
Normal weight	23	38	51	53
Overweight	18	14	11	14
Obese	13	5	4	0

**Table 3:** Analysis of body image perception by immigrant status (African-born sample *n* = 60; Italian-born sample *n* = 71).

Variables	Immigrants		Non-Immigrants		Mann–Whitney Test	
	Mean	SD	Mean	SD	*p*	Effect Size (r)
Feel figure	5.3	2.3	4.4	1.8	0.018	0.207
Ideal figure	4.5	1.9	3.5	1.2	<0.001	0.298
FID	0.77	1.63	0.89	1.41	0.623	0.043
FAI	−0.05	0.77	−0.04	0.55	0.745	0.028

Note: FID: Feel-Ideal Discrepancy Index; FAI: Feel-weight status minus Actual-weight status Inconsistency Index. FAI was calculated by comparing the BMI category derived from the selected silhouette with the participant’s BMI-based weight status category.

**Table 4:** Spearman’s rho (*ρ*) correlation coefficient between body mass index (BMI) and body image indicators (African-born sample *n* = 60; Italian-born sample *n* = 71).

Variables	Immigrants		Non-Immigrants	
	*ρ*	*p*	*ρ*	*p*
Feel figure	0.730	<0.001	0.771	<0.001
Ideal figure	0.376	0.003	0.401	0.001
FID	0.584	<0.001	0.671	<0.001
FAI	−0.248	0.056	0.034	0.779

Note: FID: Feel-Ideal Discrepancy Index; FAI: Feel-weight status minus Actual-weight status Inconsistency Index.

**Table 5:** Spearman’s rho (*ρ*) correlation coefficient between the years of residence in Italy and physical and mental health variables and behaviours (African-born sample *n* = 60; any different numbers of observations were reported in the note).

Variables	Immigrants	
	*ρ*	*p*
Age	0.403	0.002
BMI	0.155	0.242
Feel Figure	0.194	0.140
Ideal Figure	0.184	0.162
FID	0.056	0.674
FAI	0.032	0.807
RSES score ^1^	0.142	0.381
SWLS score ^2^	0.116	0.477
RAPA 1 score	−0.040	0.762
RAPA 2 score ^3^	−0.105	0.517
Mediterranean diet score ^4^	−0.150	0.369

Note: BMI: body mass index; FID: Feel-Ideal Discrepancy Index; FAI: Feel-weight status minus Actual-weight status Inconsistency Index; RSES: Rosenberg Self-Esteem Scale; SWLS: Satisfaction With Life Scale; RAPA: Rapid Assessment of Physical Activity. ^1^ African-born sample *n* = 57. ^2^ African-born sample *n* = 57. ^3^ African-born sample *n* = 55; ^4^ African-born sample *n* = 49.

**Table 6:** Analysis of RAPA according to immigrant status (RAPA 1: African-born sample *n* = 60; Italian-born sample *n* = 71; RAPA 2: African-born sample *n* = 55; Italian-born sample *n* = 71).

	Immigrants	Non-Immigrants
*RAPA 1 category*		
Sedentary	23	3
Under-active	6	7
under-active regular–light activities	4	8
Under-active regular	12	14
Active	15	39
*RAPA 2 category*		
0 (none)	40	12
1 (strength)	3	17
2 (flexibility)	9	11
3 (both)	3	31

**Table 7:** Spearman’s rho (*ρ*) correlation coefficient between the Feel-Ideal Discrepancy Index (FID) and physical and mental health variables and behaviours (African-born sample *n* = 60; Italian-born sample *n* = 71; any different numbers of observations were reported in the note).

Variables	Immigrants		Non-Immigrants	
	*ρ*	*p*	*ρ*	*p*
Self-reported weight status	0.354	0.006	0.526	<0.001
BMI-based weight status	0.569	<0.001	0.511	<0.001
RSES score ^1^	0.036	0.824	−0.001	0.997
SWLS score ^2^	−0.191	0.232	0.123	0.306
RAPA 1 score	−0.288	0.026	0.027	0.827
RAPA 2 score ^3^	−0.095	0.554	−0.243	0.042
Mediterranean diet score ^4^	−0.281	0.084	0.015	0.902

Note: BMI = body mass index; RSES: Rosenberg Self-Esteem Scale; SWLS: Satisfaction With Life Scale; RAPA: Rapid Assessment of Physical Activity. ^1^ African-born sample *n* = 57; Italian-born sample: 68. ^2^ African-born sample *n* = 57. ^3^ African-born sample *n* = 55; ^4^ African-born sample *n* = 49; Italian-born sample *n* = 67.

## Data Availability

The data that support the findings of this study are available from the corresponding author upon reasonable request. The data are not publicly available because public sharing of the full dataset was not explicitly included in the informed consent approved for this study.

## Supplementary Materials

The following supporting information can be downloaded at https://www.mdpi.com/article/10.3390/nu18152456/s1, Table S1: STROBE checklist for cross-sectional studies.

## Abbreviations

The following abbreviations are used in this manuscript:

BMI	Body Mass Index
STROBE	Strengthening the Reporting of Observational Studies in Epidemiology
ANCOVA	Analysis of covariance
SD	Standard deviation
FID	Feel-Ideal Discrepancy
FAI	Feel-weight status minus Actual-weight status Inconsistency
PASTAS	Physical Appearance State and Trait Anxiety Scale
RSES	Rosenberg Self-Esteem Scale
SWLS	Satisfaction With Life Scale
RAPA	Rapid Assessment of Physical Activity
NCD-RisC	NCD Risk Factor Collaboration
