# Supplementary material for: Body Image Perception, Weight Status and Health Behaviours Among African Immigrant Women Living in Italy: A Cross-Sectional Comparative Study
Source: Nutrients. 2026 Jul 27;18(15):2456. doi: 10.3390/nu18152456 (PMC13468556; doi:10.3390/nu18152456)
Supplement: Supplementary file 1 [file nutrients-18-02456-s001.zip › nutrients-4422801-supplementary.pdf]

Table S1: STROBE checklist for cross-sectional studies

	Item No	Recommendation	
**Title and abstract**	1	(*a*) Indicate the study's design with a commonly used term in the title or the abstract	yes-
		(*b*) Provide in the abstract an informative and balanced summary of what was done and what was found	yes
**Introduction**			
Background/rationale	2	Explain the scientific background and rationale for the investigation being reported	yes
Objectives	3	State specific objectives, including any prespecified hypotheses	yes
**Methods**			
Study design	4	Present key elements of study design early in the paper	yes
Setting	5	Describe the setting, locations, and relevant dates, including periods of recruitment, exposure, follow-up, and data collection	yes
Participants	6	(*a*) Give the eligibility criteria, and the sources and methods of selection of participants	yes
Variables	7	Clearly define all outcomes, exposures, predictors, potential confounders, and effect modifiers. Give diagnostic criteria, if applicable	yes
Data sources/ measurement	8*	For each variable of interest, give sources of data and details of methods of assessment (measurement). Describe comparability of assessment methods if there is more than one group	*yes*
Bias	9	Describe any efforts to address potential sources of bias	
Study size	10	Explain how the study size was arrived at	yes
Quantitative variables	11	Explain how quantitative variables were handled in the analyses. If applicable, describe which groupings were chosen and why	yes
Statistical methods	12	(*a*) Describe all statistical methods, including those used to control for confounding	yes
		(*b*) Describe any methods used to examine subgroups and interactions	yes
		(*c*) Explain how missing data were addressed	no
		(*d*) If applicable, describe analytical methods taking account of sampling strategy	NA
		(*e*) Describe any sensitivity analyses	no
**Results**			
Participants	13*	(a) Report numbers of individuals at each stage of study—eg numbers potentially eligible, examined for eligibility, confirmed eligible, included in the study, completing follow-up, and analysed	yes
		(b) Give reasons for non-participation at each stage	yes
		(c) Consider use of a flow diagram	no
Descriptive data	14*	(a) Give characteristics of study participants (eg demographic, clinical, social) and information on exposures and potential confounders	yes
		(b) Indicate number of participants with missing data for each variable of interest	yes
Outcome data	15*	Report numbers of outcome events or summary measures	yes

Main results	16	(*a*) Give unadjusted estimates and, if applicable, confounder-adjusted estimates and their precision (eg, 95% confidence interval). Make clear which confounders were adjusted for and why they were included	yes
		(*b*) Report category boundaries when continuous variables were categorized	yes
		(*c*) If relevant, consider translating estimates of relative risk into absolute risk for a meaningful time period	NA
Other analyses	17	Report other analyses done—eg analyses of subgroups and interactions, and sensitivity analyses	yes
**Discussion**			
Key results	18	Summarise key results with reference to study objectives	yes
Limitations	19	Discuss limitations of the study, taking into account sources of potential bias or imprecision. Discuss both direction and magnitude of any potential bias	yes
Interpretation	20	Give a cautious overall interpretation of results considering objectives, limitations, multiplicity of analyses, results from similar studies, and other relevant evidence	yes
Generalisability	21	Discuss the generalisability (external validity) of the study results	yes
**Other information**			
Funding	22	Give the source of funding and the role of the funders for the present study and, if applicable, for the original study on which the present article is based	NA

*Give information separately for exposed and unexposed groups.

Note: An Explanation and Elaboration article discusses each checklist item and gives methodological background and published examples of transparent reporting. The STROBE checklist is best used in conjunction with this article (freely available on the Web sites of PLoS Medicine at http://www.plosmedicine.org/, Annals of Internal Medicine at http://www.annals.org/, and Epidemiology at http://www.epidem.com/). Information on the STROBE Initiative is available at www.strobe-statement.org.
